# Single-Cell RNA Sequencing of Peripheral Blood Mononuclear Cells From Acute Myocardial Infarction

**DOI:** 10.3389/fimmu.2022.908815

**Published:** 2022-06-29

**Authors:** Jun Qian, Yanhua Gao, Yan Lai, Zi Ye, Yian Yao, Keke Ding, Jing Tong, Hao Lin, Guoqi Zhu, Yunan Yu, Haoran Ding, Deqiang Yuan, Jiapeng Chu, Fei Chen, Xuebo Liu

**Affiliations:** Department of Cardiology, Tongji Hospital, Tongji University School of Medicine, Shanghai, China

**Keywords:** plaque rupture, plaque erosion, single-cell RNA sequencing, acute myocardial infarction, inflammatory

## Abstract

**Background:**

Acute myocardial infarction (AMI) can occur in patients with atherosclerotic disease, with or without plaque rupture. Previous studies have indicated a set of immune responses to plaque rupture. However, the specific circulating immune cell subsets that mediate inflammatory plaque rupture remain elusive.

**Methods:**

Ten AMI patients were enrolled in our study (five with and five without plaque rupture; plaque characteristics were identified by optical coherence tomography). By single-cell RNA sequencing, we analyzed the transcriptomic profile of peripheral blood mononuclear cells.

**Results:**

We identified 27 cell clusters among 82,550 cells, including monocytes, T cells, NK cells, B cells, megakaryocytes, and CD34^+^ cells. Classical and non-classical monocytes constitute the major inflammatory cell types, and pro-inflammatory genes such as CCL5, TLR7, and CX3CR1 were significantly upregulated in patients with plaque rupture, while the neutrophil activation and degranulation genes FPR2, MMP9, and CLEC4D were significantly expressed in the intermediate monocytes derived from patients without plaque rupture. We also found that CD4^+^ effector T cells may contribute to plaque rupture by producing a range of cytokines and inflammatory-related chemokines, while CD8^+^ effector T cells express more effector molecules in patients without plaque rupture, such as GZMB, GNLY, and PRF1, which may contribute to the progress of plaque erosion. Additionally, NK and B cells played a significant role in activating inflammatory cells and promoting chemokine production in the plaque rupture. Cell–cell communication elaborated characteristics in signaling pathways dominated by inflammatory activation of classical monocytes in patients with plaque rupture.

**Conclusions:**

Our studies demonstrate that the circulating immune cells of patients with plaque rupture exhibit highly pro-inflammatory characteristics, while plaque erosion is mainly associated with intermediate monocyte amplification, neutrophil activation, and degranulation. These findings may provide novel targets for the precise treatment of patients with AMI.

## Introduction

Acute myocardial infarction (AMI) is currently the primary cause of disability, death, and loss of quality of life (1). Autopsy studies indicate that plaque rupture is the dominating pathological mechanism of AMI. However, the gradual strengthening of treatments, such as lowering blood pressure or lipids, and smoking cessation, gradually increased the AMI caused by plaque erosion (2). Additionally, the thrombosis caused by plaque erosion has a different mechanism than plaque rupture (3). The plaques formed by superficial erosion often have fewer lipids, are rich in matrix, have an intact fibrous cap and abundant platelets, and lack obvious aggregation of macrophages, while the plaques caused by rupture normally have large lipid pools, thin ruptured fiber caps, and are rich in foam cells (4).

Intravascular imaging techniques, such as optical coherence tomography (OCT), make it possible to identify plaque characteristics *in vivo*. Due to the invasiveness and high cost of intraluminal imaging techniques such as OCT, it is unrealistic to solely rely on this method to classify AMI. Treatment methods differ based on the imaging manifestations of plaque rupture and erosion. The OCT-based EROSION study suggests that AMI caused by plaque erosion does not require stent implantation but only medical treatment (5). Previous studies have not thoroughly explored the circulating immune molecular differences and pathological mechanisms of plaque rupture and erosion.

Given the limited molecular mechanisms and pathophysiology of plaque rupture and erosion, developing novel blood biomarkers using genetics and transcriptomics to identify plaque rupture or erosion may revolutionize the early diagnosis and treatment of AMI. Here, we performed single-cell RNA sequencing to accurately dissect the cellular immunological landscape of peripheral blood in AMI patients. In this study, we identified several key cell subsets and hub genes that contribute to the progression of plaque rupture and erosion, which may provide potential therapeutic targets for cardiovascular events.

## Methods

### Study Design and Population

A total of 20 patients with AMI who underwent percutaneous coronary intervention (PCI) at the Shanghai Tongji Hospital from April to December 2020 were included in the study. AMI was classified as STEMI and non-ST segment elevation MI (NSTEMI). The STEMI diagnosis was based on typical chest pain and new ST-segment elevation of at least two contiguous leads >0.1 mV or new left bundle-branch block on the 12-lead electrocardiogram (ECG) and elevated cardiac markers (troponin T/I or creatine kinase-MB) (6). On the 12-lead ECG, NSTEMI was identified as persistent angina accompanied by elevated cardiac markers but without the ST-segment elevation. Exclusion criteria were a left ventricular ejection fraction <30%; severe infectious disease, sepsis, or autoimmune disease; or malignant tumor or life expectancy of <1 year. All patients gave written informed consent, and the ethics committee of the Shanghai Tongji Hospital approved the study protocol. This research was in line with the Declaration of Helsinki.

### OCT Procedure and Analysis

All patients underwent coronary angiography *via* the transradial or transfemoral approach. We confirmed the culprit lesion by ST-segment changes in ECG, coronary angiography, and echocardiography. All patients received a loading dose of aspirin at 300 mg and ticagrelor at 180 mg, followed by oral aspirin at 100 mg once daily and ticagrelor at 90 mg twice daily. We performed OCT using the frequency domain (C7-XR, OCT intravascular imaging system; St. Jude Medical) OCT system. Previous studies have described detailed techniques for OCT imaging in intracoronary (7, 8).

All OCT images were analyzed by two independent researchers who were ignorant of the angiography, clinical, and laboratory data. Any inconsistencies were resolved through consultation with a third auditor. Based on the previously established OCT diagnostic criteria, plaque in the culprit lesions was divided into three categories: plaque fibrous cap rupture (PR), non-plaque fibrous cap rupture (NPR), and calcified nodules or others. The PR was determined by the discontinuous fiber cap, where the plaque cavity and the inner core of the plaque were connected or the cavity was formed (9, 10). In this definition, NPR was defined as the detectable thrombus covering the plaque surface without ruptured fibrous cap or the irregular luminal surface, which in the absence of the thrombus corresponds to plaque erosion (11–13). NPR was an exclusionary diagnosis that required non-rupture of the fibrous cap.

### Biosample Collection and Storage

We continuously collected peripheral arterial blood samples of patients with PR and NPR before coronary angiography within six hours of symptom onset. The samples were collected in EDTA-coated tubes before heparin or any contrast agent was administered.

#### Single-Cell RNA Preparation and Sequencing

We processed all blood samples within 2 h of collection. We used a Ficoll–Plaque medium to separate peripheral blood mononuclear cells (PBMCs). Cell viability exceeded 90% as determined by trypan blue staining. Chromium Next GEM Single Cell 3’ v3 (10× Genomics) was used for single cell capture and library construction according to the specifications of the manufacturer. Sequencing was performed on the Illumina NovaSeq platform.

### Single-Cell RNA Sequencing Data Analysis

Single-cell expression data, including de-multiplexing, genome alignment (GRCh38), barcode counting, and unique molecular identifier (UMI) counting, were processed using the Cell Ranger pipeline (v3.0.1, 10× Genomics). Seurat (V3.0.2) was used to analyze the gene barcode matrix and data integration of UMI counting (14). This output was then imported into Seurat (v3.0.2) R for subsequent quality control and downstream analysis of single-cell RNA sequencing data (15). The PercentFeatureSet function of the Seurat software package was used to calculate the mitochondrial gene expression (16). Cells with >25% UMIs from the mitochondrial genome were removed through quality control. The integrated matrix was then scaled and the first 30 dimensions from principal component analysis (PCA) were used for t-distributed stochastic neighbor embedding (t-SNE) visualization. We applied the same procedure of scaling, dimensionality reduction, and clustering to the specific data sets of sub-clustering. We used the Wilcoxon rank-sum test to find significantly differentially expressed genes (DEGs) in each cluster by comparing other clusters. SingleR and cardinal marker genes were used to identify cell types (17). The Top100 significantly upregulated DEGs were imported into the STRING website (http://string-db.org/) for further analysis to screen hub differential genes in Cytoscape software. Generally, the genes with the most connections are the most significant genes in a module. Hub genes are called “highly linked genes” and tend to have high links in a co-expressed module. Protein–protein interaction (PPI) networks were constructed using the Cytoscape software. ClusterProfiler (v3.14.0) was used for functional enrichment analyses. Gene Ontology (GO), Kyoto Encyclopedia of Genes and Genomes (KEGG), and Gene Set Enrichment analysis (GSEA) pathway enrichment analysis of DEGs were performed by ClusterProfiler (v 3.14.0) package.

### Pseudo-Time Trajectory and SCENIC Analysis

The R package monocle2 (v2. 14. 0) (18) was used to identify trajectories of single-cell to detect cell state transitions. Single Cell Regulatory Network Inference and Clustering (SCENIC) analysis was performed as previously described (19). We used the SCENIC package (v1.2.2), a SCENIC pipeline that is quickly implemented in Python.

### Cell–Cell Communication Analysis

We used the CellChat package based on the R language for the analysis of cell–cell communication (20). Overexpressed ligands or receptors identified in cell populations are projected into the PPI network. CellChat extrapolates biologically important cell–cell communication by assigning a probability value to each interaction and performing permutation tests. The circle and bubble graphs were used to visualize the communication network and signal path.

### Statistical Analysis

Statistical analyses were performed using the R (version 3.6.1; http://www.R-project.org, R Foundation for Statistical Computing, Vienna, Austria) and Python software. Categorical variables are expressed in counts and proportions (%). The data were tested for normal distribution using the Kolmogorov–Smirnov test. Continuous variables are expressed as mean ± SD. Comparison between groups of continuous variables was performed by Student’s t-test or Mann–Whitney *U* test, and the chi-square test or Fisher’s exact test was used for categorical results. The significance level was set at a two-sided α value of 0.05.

## Results

### Baseline Characteristics

The study flowchart is presented in **Supplementary Figure 1**. We excluded nine patients due to refusal to enroll (n = 4), inability to obtain suitable images (n = 3), and predilation due to heavy stenosis (n = 2). Finally, eleven patients underwent OCT imaging, with PR identified in five, NPR in five, and one was undefined. A total of 10 patients presenting with AMI were enrolled in the single-cell RNA sequencing. There were no differences in baseline characteristics between patients with and without PR (**Supplementary Table 1**). Representative coronary angiography and OCT images are shown in **Supplementary Figure 2**.

### Circulating Immune Cell Landscape in Patients With and Without PR

**Figure 1:**
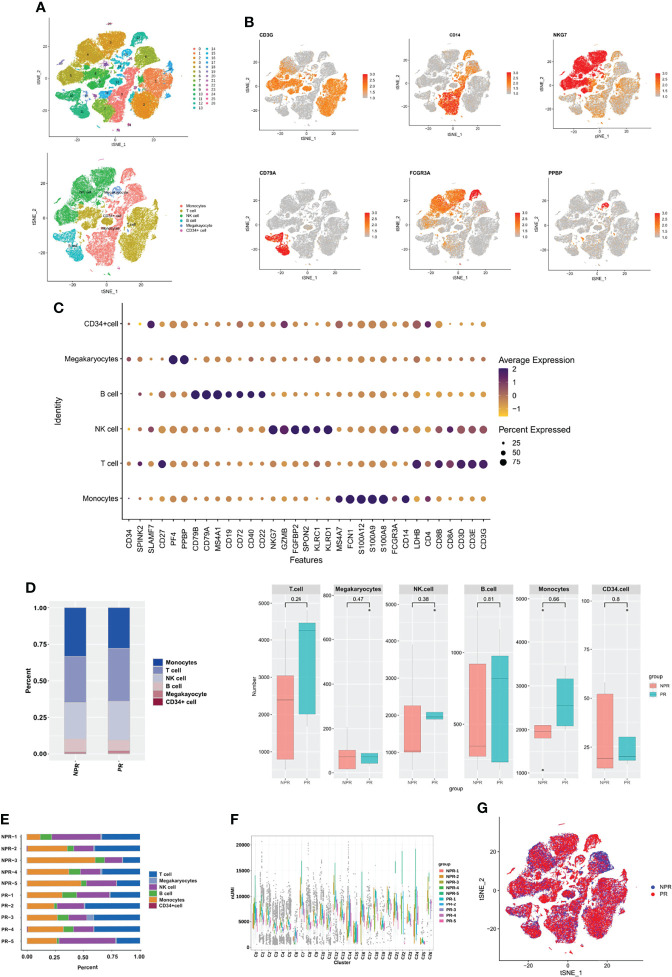
Circulating immune cell landscape in patients with and without PR. **(A)** t-SNE plot presenting clustering of total PBMCs reveals 27 clusters and 6 cell types. **(B)** t-SNE plot of marker genes of each cell type. **(C)** Dot plot showing the top marker genes revealed by single-cell RNA sequencing of cell types defined in **(A)**. **(D)** Percentage of cell types and the comparison of cell number in each cell type between PR and NPR. **(E)** Cell proportion of each patient. **(F)** UMI number of each cluster. **(G)** t-SNE plot of patients with PR and NPR.

After filtering cells with low RNA content or high mitochondrial RNA, we annotated a total of 82,550 cells (3,433–14,504 per patient). Unsupervised organization of gene expression revealed 27 clusters and 6 cell types (**Figure 1A**). The estimated number of cells, mean reads per cell, median genes per cell, and cell viability before loading of each patient are presented in **Supplementary Table 2**. Marker genes for each cell type are shown in **Figures 1B, C**. T cells (28,195, 34.16%) constituted the major population of immune cells in patients with AMI, followed by monocytes (24,910, 30.18%), NK cells (21,348, 25.86%), B cells (6,383, 7.73%), megakaryocytes (1,379, 1.67%), and CD34^+^ cells (335, 0.40%). No significant differences were detected when comparing the percentage of cells derived from patients with and without PR (**Figure 1D**). The cell composition ratio of each patient, the number of UMI for each cluster, and the t-SNE plot of the two groups are shown in **Figures 1E–G**.

### Circulating Monocytes in Patients With PR Showed a More Active Inflammatory State

**Figure 2:**
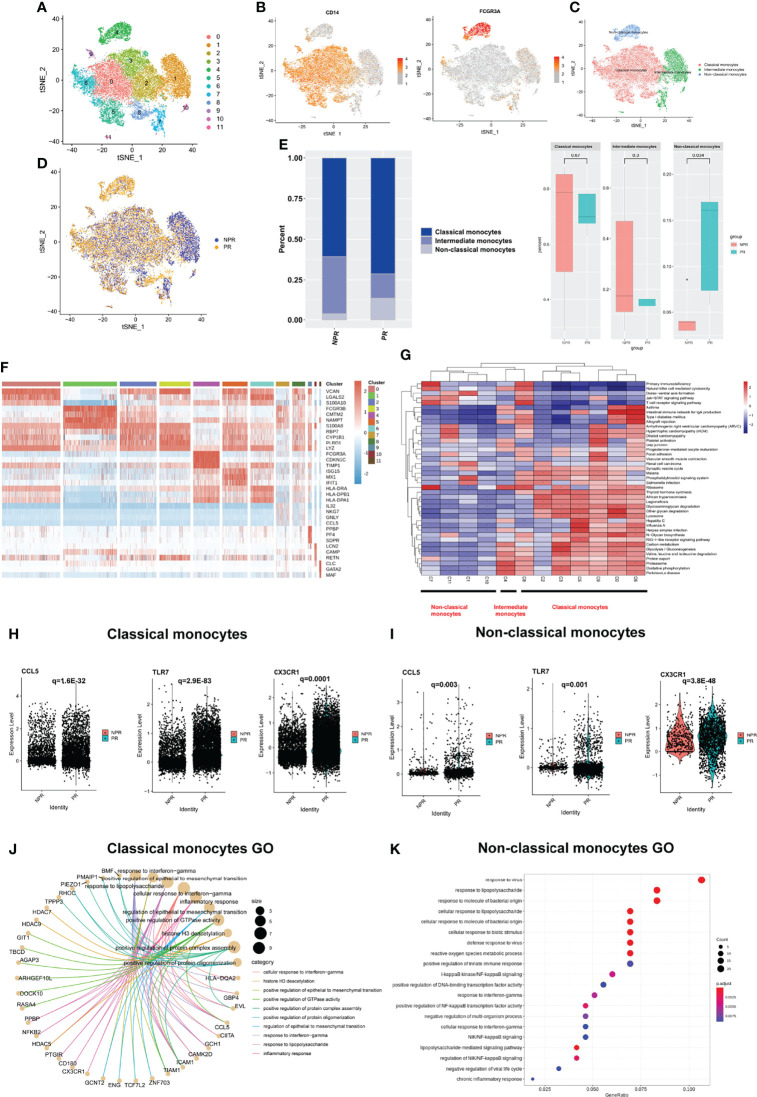
Characterizations of circulating monocytes in patients with and without plaque rupture. **(A)** t-SNE plot showing re-clustering of monocyte cells into 12 clusters. **(B)** Expression t-SNE plot of monocytes markers CD14 and FCGR3A (CD16). **(C)** t-SNE plot of monocytes divided into three cell types: classical, intermediate, and non-classical monocytes. **(D)** t-SNE plot showing contribution of monocytes for patients with PR and NPR. **(E)** Percentage of monocytes subtype in patients with PR and NPR. **(F)** Heatmap showing expression levels of discriminative genes for each cluster. **(G)** GSEA heatmap of total cluster in monocytes. **(H, I)** Prototypical pro-inflammatory hub genes such as CCL5, TLR7, and CX3CR1 identified by STRING and Cytoscape were significantly upregulated in classical and non-classical monocytes derived from patients with PR. **(J)** Top GO terms of classical monocytes in patients with PR. **(K)** Top GO terms of non-classical monocytes in patients with PR.

Further annotation of the monocyte clusters in the initial annotation based on CD14 and FCGR3A yielded three types: classical (CD14 high, clusters 0, 2, 3, 5, 6, 8, and 9); intermediate (CD14 low, FCGR3A(CD16) low, clusters 1, 7, 10, and 11); and non-classical monocytes (CD14 low, FCGR3A(CD16) high, and cluster 4) (**Figures 2A–C** and **Supplementary Figure 3**). The t-SNE plot of monocytes derived from PR and NPR is shown in **Figure 2D**. Classical monocytes accounted for 71.3% of the monocytes in patients with PR and 60.7% of the monocytes in patients without PR (p = 0.67; **Figure 2E**). Intermediate monocytes accounted for a lower proportion of monocytes in patients with PR (15.0%) vs. NPR (35.5%), although the difference did not reach statistically significance (p = 0.3; **Figure 2E**). The proportion of non-classical monocytes was higher in patients with PR (13.7%) than in those without PR (3.8%) (p = 0.034; **Figure 2E**). The marker genes of each cluster are presented in **Figure 2F** and **Supplementary Figure 3**.

**Figure 3:**
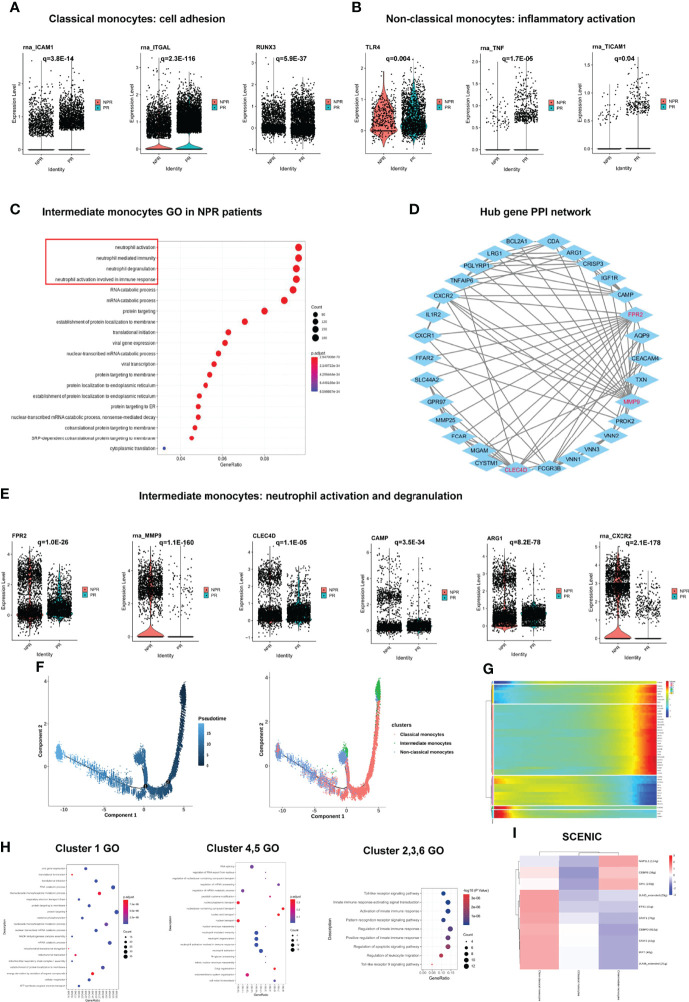
Impact of plaque rupture and without rupture on gene regulation and development trajectory of circulating monocytes. **(A)** Expression of prototypical leucocyte-endothelial cell adhesion genes such as ICAM1, ITGAL, and RUNX3 were significant upregulated in classical monocytes of patients with PR. **(B)** Expression of prototypical pro-inflammatory proteins such as TLR4, TNF, and TICAM1 were significantly expression in non-classical monocytes of patients with PR. **(C)** GO analysis of upregulated genes in intermediate monocytes derived from patients with NPR. **(D)** The PPI network of hub upregulated genes in intermediate monocytes in patients with NPR. **(E)** Significantly upregulated hub genes associated with neutrophil activation and degranulation in intermediate monocytes derived from patients with NPR. **(F)** The developmental trajectories of different monocyte subtypes with pseudo-time analysis. **(G)** Heatmap of significantly upregulated genes in pseudo-time developmental trajectories. **(H)** GO analysis of upregulated genes in different clusters in pseudo-time analysis. **(I)** Heatmap of upstream transcription factors in monocytes.

GSEA of the three types of monocytes showed distinct characteristics. Classical monocytes were mainly enriched in “glycosaminoglycan degradation” and “lysosome and oxidative phosphorylation,” whereas non-classical monocytes were mainly enriched in “Jak−STAT signaling pathway” and “T-cell receptor signaling pathway” (**Figure 2G**). We took the intersection of the top100 upregulated DEGs in three types of monocytes from patients with PR to find the common Hub genes. Results showed that there were 20 common Hub genes in the classical and non-classical monocytes but only 2 common genes in all three types (**Supplementary Figure 4**), indicating that intermediate monocytes have distinct transcription characteristics from others. We imported the common genes of classical and non-classical monocytes into STRING and Cytoscape for further analysis (**Supplementary Figure 4**). We found that prototypical pro-inflammatory hub genes such as CCL5, TLR7, and CX3CR1 identified by STRING and Cytoscape were all significantly upregulated in patients with PR from classical and non-classical monocytes (**Figures 2H, I**). Assessment of the top GO terms, including “inflammatory response” and “positive regulation of NF-kappaB signaling,” further identified inflammatory molecules that were observably increased in classical and non-classical monocytes derived from patients with PR (**Figures 2J, K**). Leucocyte-endothelial cell adhesion genes such as ICAM1, ITGAL, and RUNX3 were also significantly expressed in classical monocytes derived from patients with PR, implying a series of interactions between classical monocytes and endothelial cells (**Figure 3A**). Considering that non-classical monocytes accounted for a high proportion of PR, we further analyzed hub genes that were upregulated in non-classical monocytes from patients with PR. The significant upregulated hub genes in non-classical monocytes, including TLR4, TNF, and TICM1, further validated that patients with PR presented as a more inflammatory activation state than NPR (**Figure 3B**).

In contrast to the classical and non-classical monocytes, intermediate monocytes accounted for a higher percentage of patients with NPR. The top GO terms were associated with “neutrophil degranulation,” “neutrophil activation,” and “neutrophil mediate immunity” in intermediate monocytes derived from patients with NPR (**Figure 3C**). For example, FPR2, MMP9, CLEC4D, CAMP, ARG1, and CXCR2, the neutrophil activation and degranulation genes, were profoundly upregulated in intermediate monocytes derived from patients with NPR (**Figures 3D, E**). Previous studies reported that neutrophil activation and degranulation promoted the production of neutrophil extracellular traps (NETs), which may contribute to plaque erosion (21, 22).

To identify transcriptional characteristics at different stages of monocyte development, we conducted a pseudo-time analysis to identify trajectories of gene expression associated with functional changes. We found that classical and intermediate monocytes were mainly at the beginning of the developmental trajectory, whereas non-classical monocytes were primarily at the end of the pseudo-temporal trajectory (**Figure 3F**). We divided the monocytes in the developmental trajectory into 6 clusters (**Figure 3G**). Classical monocytes (referring to cells in cluster 1) showed ontology terms such as “translational initiation” and “mRNA catabolic process,” which indicates metabolism involvement (**Figure 3H**). Intermediate monocytes (referring to cells in clusters 4 and 5) exhibited GO terms such as “neutrophil activation and degranulation” and “neutrophil activation involved in immune response,” which further corroborates previous analysis (**Figure 3H**). Non-classical monocytes (referring to clusters in 2, 3, and 6) displayed ontology terms such as “toll-like receptor signaling pathway” and “activation of innate immune response,” which were also presented as a highly proinflammatory state (**Figure 3H**). Pseudo-temporal analysis showed that the peripheral circulating monocytes of patients with AMI indicated a gradual transition from classical and intermediate monocytes to non-classical monocytes.

We then applied SCENIC analysis to identify TFs with gene expression differences among the monocytes. Of note, the gene expression regulated by NAP1L1, CEBPB, and SPI1 was upregulated in intermediate monocytes. The expression of STAT1, STAT2, JUNB, JUND, IRF7, ETS1, and CEBPD was prominent in non-classical monocytes, which were mainly associated with “response to cytokine,” “type I interferon signaling pathway,” and “cytokine-mediated signaling pathway” (**Figure 3I**). These results imply that TFs in non-classical monocytes may facilitate the inflammatory process in PR.

#### CD4^+^ Effector T Cells and CD8^+^ Effector T Cells Showed Completely Distinct Characteristics

**Figure 4:**
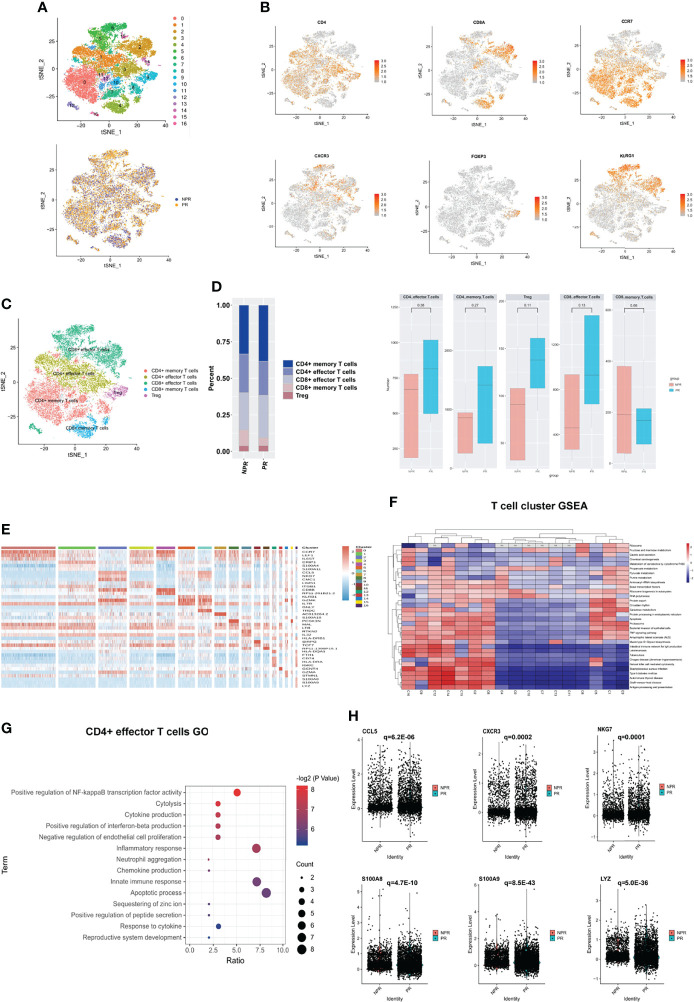
Characterizations of T cells in patients with and without plaque rupture. **(A)** t-SNE plot showing re-clustering of T cells into 17 clusters and t-SNE plot of patients with PR and NPR. **(B)** Expression t-SNE plot of T cell marker genes as CD4, CD8A, CCR7, CXCR3, FOXP3 and KLRG1. **(C)** Percentage of T cell subtype in patients with PR and NPR. **(D)** t-SNE plot showing the contribution of different T cell subsets. **(E)** Heatmap showing expression levels of discriminative genes for each cluster. **(F)** GSEA heatmap of T cell clusters. **(G)** GO analysis of upregulated genes in CD4^+^ effector T cells from patients with PR. **(H)** Expression of pro-inflammatory hub genes such as CCL5, CXCR3, NKG7, S100A8, S100A9, and LYZ were significantly upregulated in CD4^+^ effector T cells of patients with PR.

A sub-clustering of T cells identified 17 clusters. We separated T cell clusters into CD4^+^ and CD8^+^ cells and further subdivided these into CD4^+^ effector T cells (clusters 1 and 3), CD4^+^ memory T cells (clusters 0, 7, 8, 10, 11, 13, and 16), CD4^+^ regulatory T cells (Tregs, cluster 9), CD8^+^ memory T cells (cluster 4), and CD8^+^ effector T cells (clusters 2, 5, 6, 12, 14, and 15) (**Figures 4A–C**). The expression of additional prototypical marker genes is shown in **Figure 4B** and **Supplementary Figure 5**. CD4^+^ memory T cells and CD8^+^ memory T cells were identified by significantly expressed marker genes such as CCR7, while cytotoxic markers such as KLRG1 were highly increased in CD8^+^ effector T cells (**Figures 4B, C**). There was no significant difference in the percentage of CD4^+^ and CD8^+^ T cells between patients with PR and NPR (**Figure 4D**).

**Figure 5:**
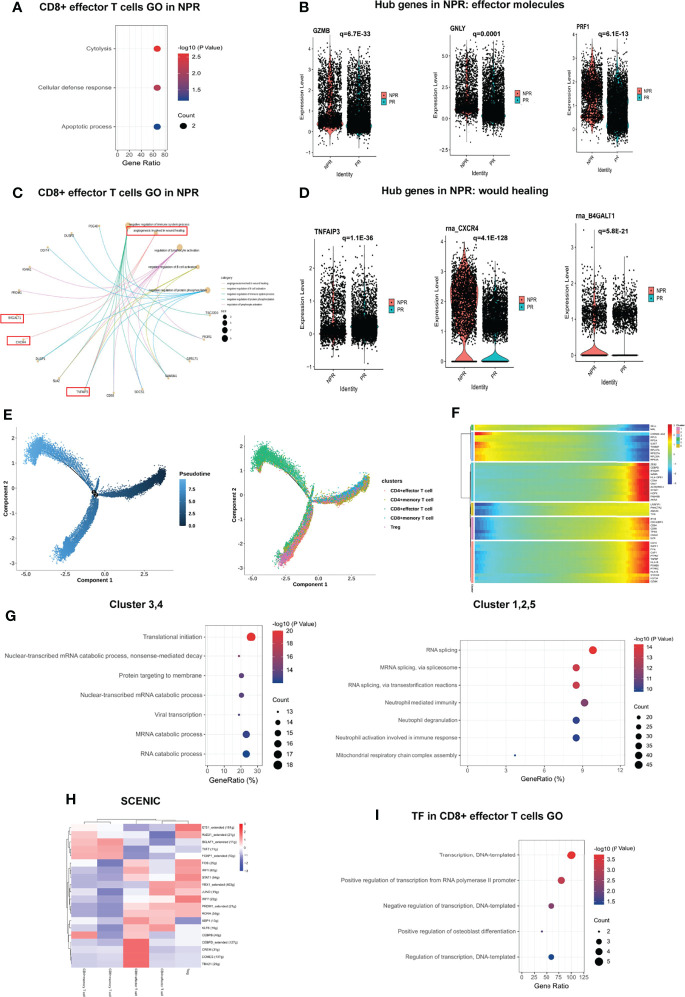
Impact of plaque rupture and without rupture on gene regulation and development trajectory of circulating T cells. **(A, B)** Expression of effector molecules associated with apoptosis and desquamation of endothelial cells as GZMB, GNLY, and PRF1 in CD8^+^ effector T cells of patients with NPR. **(C, D)** Expression of angiogenesis and wound healing genes as TIFAIP3, CXCR4, and B4GALT1 in CD8^+^ effector T cells of patients with NPR. **(E)** The developmental trajectories of different T cell subtypes with pseudo-time analysis. **(F)** Heatmap of significantly upregulated genes in pseudo-time developmental trajectories. **(G)** GO analysis of upregulated genes in different clusters in a pseudo-time analysis. **(H)** Heatmap of upstream transcription factors in T cell subtypes. **(I)** GO analysis of transcription factors in CD8^+^ effector T cells.

**Figure 4E** shows the expression of marker genes in each sub-cluster in detail. The results of the GSEA analysis showed that CD4^+^ T cells were mainly enriched in “fructose and mannose metabolism,” while CD8^+^ T cells were mainly enriched in “apoptosis,” “TNF signaling pathway,” and “antigen processing and presentation” (**Figure 4F**). The top GO analyses of CD4^+^ effector T cells derived from patients with PR were mainly enriched in the “positive regulation of NF-kappaB transcription factor activity,” “chemokine and cytokine production,” and “inflammatory response” (**Figure 4G**). Additionally, several pro-inflammatory hub proteins, such as CCL5, CXCR3, NKG7, S100A8, S100A9, and LYZ, were significantly upregulated in PR from CD4+ effector T cells, which has confirmed that these genes may contribute to plaque rupture (**Figure 4H** and **Supplementary Figure 6A**) (23–25). CD8^+^ effector T cells significantly released more effector-associated molecules in patients with NPR, such as GZMB, GNLY, and PRF1, which have been confirmed to promote the apoptosis and desquamation of endothelial cells (**Figures 5A, B** and **Supplementary Figure 6B**) (26). Up-regulated differential genes, such as TNFAIP3, CXCR4, and B4GALT1 in CD8^+^ effector T cells in patients with NPR were mainly involved in “angiogenesis and wound healing,” which may promote plaque healing in patients with NPR (**Figures 5C, D**) (27).

Development trajectory analysis of CD4^+^ and CD8^+^ T cells suggests a trajectory that starts with the Tregs, CD4^+^ memory, and CD8^+^ memory T cells and bifurcates into either CD4^+^ effector T cells or CD8^+^ effector T cells (**Figure 5E**). Notably, we observed higher expression of SELL and MAL at the beginning of the developmental trajectory, while CD8A, GZMA, GNLY, GZMK, and S100A9 were prominently expressed in CD8^+^ effector T cells at the end of the trajectory (**Figure 5F**). Memory T cells (referring to clusters 3 and 4) at the beginning of the trajectory were mainly enriched in “translational initiation,” “nuclear-transcribed mRNA catabolic process” and “mRNA catabolic process,” while CD8^+^ effector T cells (referring to clusters 1, 2, and 5) at the root of the trajectory were mainly enriched in “RNA splicing,” “neutrophil degranulation,” and “neutrophil activation involved in immune response” (**Figure 5G**).

SCENIC analysis reveals the underlying TFs as the basis for the regulation across subtypes. Interestingly, CD4^+^ memory T cells shared an upregulated expression pattern of TFs with CD8^+^ memory T cells, such as BCLAF1, TCF7, and FOXP1, implying the potential roles of these TFs in regulating the translational initiation in the biological process (**Figure 5H**). Previous studies have indicated that TCF7 and FOXP1 contribute to the development and homeostasis of memory T cells (28, 29). CD4^+^ effector and CD8^+^ effector T cells also shared some upregulated TFs, including PRDM1, RORA, and KLF6, while CEBPB, CEBPD, CREM, EOMES, and TBX21 were upregulated only in CD8^+^ effector T cells, which were mainly associated with “regulation of transcription” (**Figure 5I**).

### Characterizations of NK Cells in Patients With and Without PR

**Figure 6:**
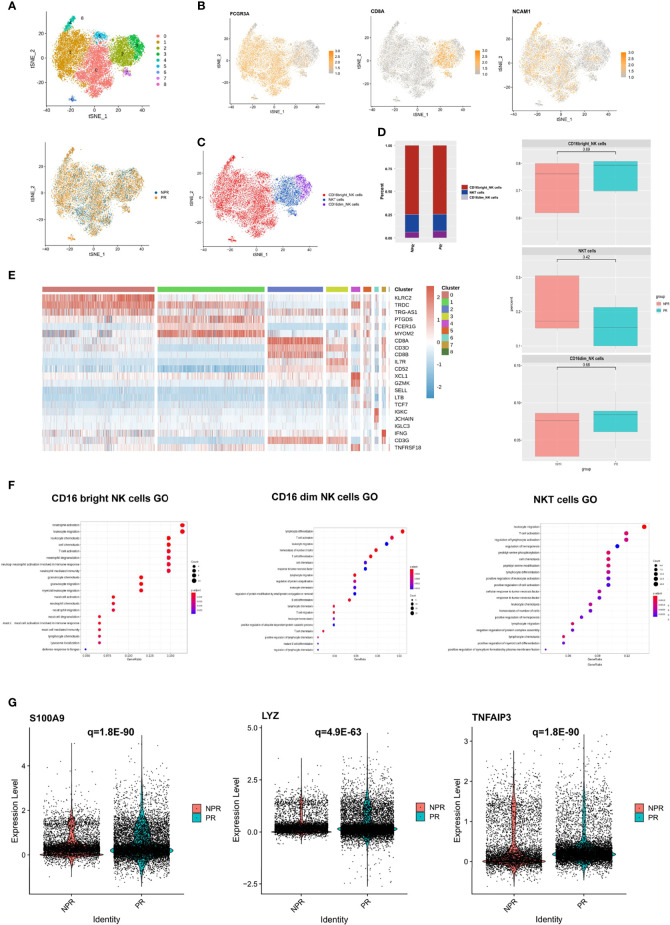
Characterization of NK cells in patients with and without plaque rupture. **(A)** t-SNE plot showing the re-clustering of NK cells into 9 clusters and t-SNE plot of patients with PR and NPR. **(B)** Expression t-SNE plot of NK cell marker genes as FCGR3A, CD8A, and NCAM1. **(C)** t-SNE plot showing the contribution of different NK cell subsets. **(D)** Percentage of NK cell subtypes in patients with PE and PR. **(E)** Heatmap showing expression levels of discriminative genes for each cluster. **(F)** GO analysis of upregulated genes in CD16^bright^ NK, CD16^dim^ NK, and NKT cells. **(G)** Expression of pro-inflammatory hub genes such as S100A9, LYZ, and TNFAIP3 were significantly upregulated in CD16^bright^ NK cells of patients with PR.

A total of 21,348 NK cells were re-clustered into 9 clusters. These clusters were annotated as CD16^bright^ NK cells (clusters 0, 1, 4, 5, 6, and 8), CD16^dim^ NK cells (cluster 3), and NKT cells (clusters 2 and 7) (**Figure 6A**). CD16^bright^ NK cells were positive for FCGR3A (CD16) and CD8A was the marker gene of NKT cells (**Figures 6B, C**). There was no difference regarding cell percentage in the three types of NK cells between PR and NPR patients (**Figure 6D**). The marker gene heatmap of each cluster of NK cells is shown in **Figure 6E** and **Supplementary Figure 7**. In GO terms, CD16^bright^ NK, CD16^dim^ NK, and NKT cells derived from patients with PR were mainly enriched in “leukocyte chemotaxis and migration” and “neutrophil activation,” implying that NK cells increased the accumulation of inflammatory cells in patients with PR (**Figure 6F**). Upregulated differentially pro-inflammatory hub genes, such as S100A9, LYZ, and TNFAIP3, were also markedly expressed in CD16^bright^ NK cells derived from patients with PR (**Figure 6G**).

Nevertheless, the GO terms of all NK cell types of patients with NPR were mainly enriched in “negative regulation of leukocyte and lymphocyte activation and the immune system,” indicating that NK cells played a negatively regulate role in NPR (**Supplementary Figure 8A**). For example, prototypical genes that negative regulation of the immune system, such as CEBPB, TNFAIP3, and SOCS1, were significantly expressed in NK cells derived from patients with NPR. Additionally, GO terms of CD16^dim^ NK and NKT cells of NPR were enriched in “angiogenesis involved in wound healing,” which was consistent with CD8+ effector T cells in patients with PE (**Supplementary Figures 8B, C**). Together, NK cells of patients with PR presented significant activation of inflammatory cells, while patients with NPR showed negative regulation of the immune system and cells.

### B Cells Play a Centricity Role in Producing Inflammatory Cytokines

**Figure 7:**
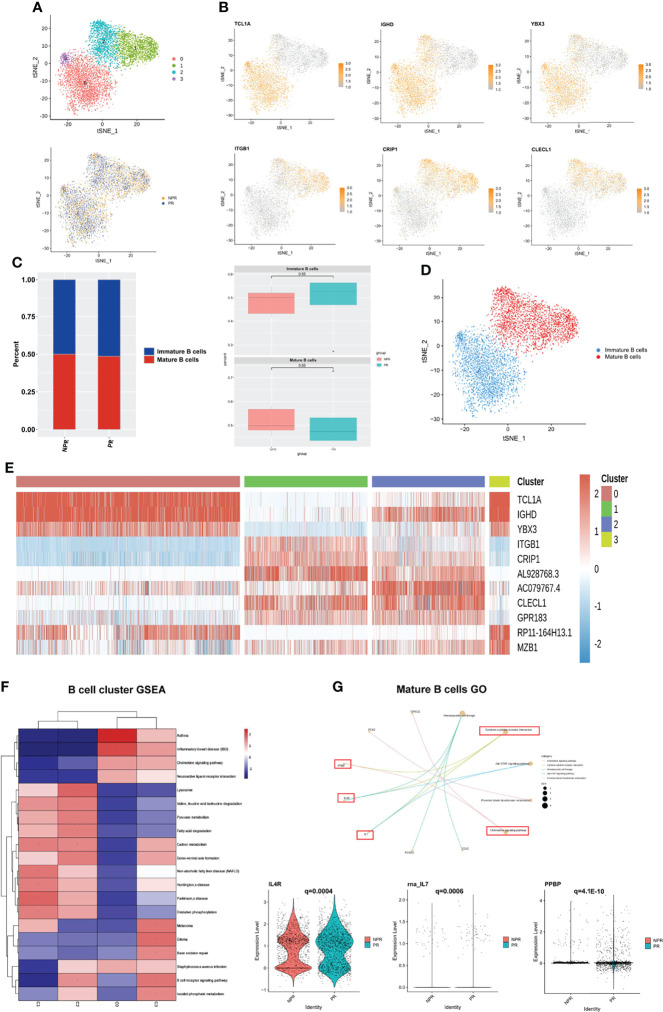
B cells play an important role in producing inflammatory cytokines. **(A)** t-SNE plot showing re-clustering of B cells into 4 clusters and t-SNE plot of patients with PR and NPR. **(B)** Expression t-SNE plots of B cell marker genes as TCL1A, IGHD, YBX3, ITGB1, CRIP1, and CLEC1. **(C)** Percent of B cell subtypes in patients with PR and NPR. **(D)** t-SNE plot showing the contribution of different B cell subsets. **(E)** Heatmap showing expression levels of discriminative genes for each cluster. **(F)** GSEA heatmap of B cell clusters. **(G)** GO analysis of mature B cells and IL4R, IL7, and PPBP were significant upregulated in patients with PR.

We detected 6,383 B cells that were re-clustered into 4 clusters. These clusters were divided into two groups: immature B cells (clusters 0 and 3) and mature B cells (clusters 1 and 2) (**Figures 7A, B**). Immature B cells expressed TCL1A, IGHD, and YBX3 at high levels, whereas mature B cells expressed ITGB1, CRIP1, and CLEC1 at high levels (**Figure 7B**). This finding suggests that immature B cells are more “silent” and mature B cells more “active.” There was no difference in the percentage of immature and mature B cells between patients with PR and NPR (**Figures 7C, D**). Marker genes in each cluster of B cells are shown in **Figure 7E** and **Supplementary Figure 9**. The GSEA analysis of immature B cells included “chemokine signaling pathway” and “neuroactive ligand−receptor interaction,” while the GSEA of mature B cells included “lysosome,” “fatty acid degradation,” and “oxidative phosphorylation” (**Figure 7F**). The top GO terms of mature B cells in patients with PR were “chemokine signaling pathway” and “cytokine–cytokine receptor interaction,” with IL4R, IL7, and PPBP being significantly upregulated and associated with these biological processes (**Figure 7G**). Additionally, GO terms of mature B cells of NPR patients were enriched in “antigen receptor-mediated signaling pathway” and “immune response-activating cell surface receptor signaling pathway,” indicating that mature B cells are mainly associated with producing inflammatory cytokines (**Supplementary Figure 10**).

### Complex Intercellular Communication Network in the Circulating Immune Cells in Patients With AMI

**Figure 8:**
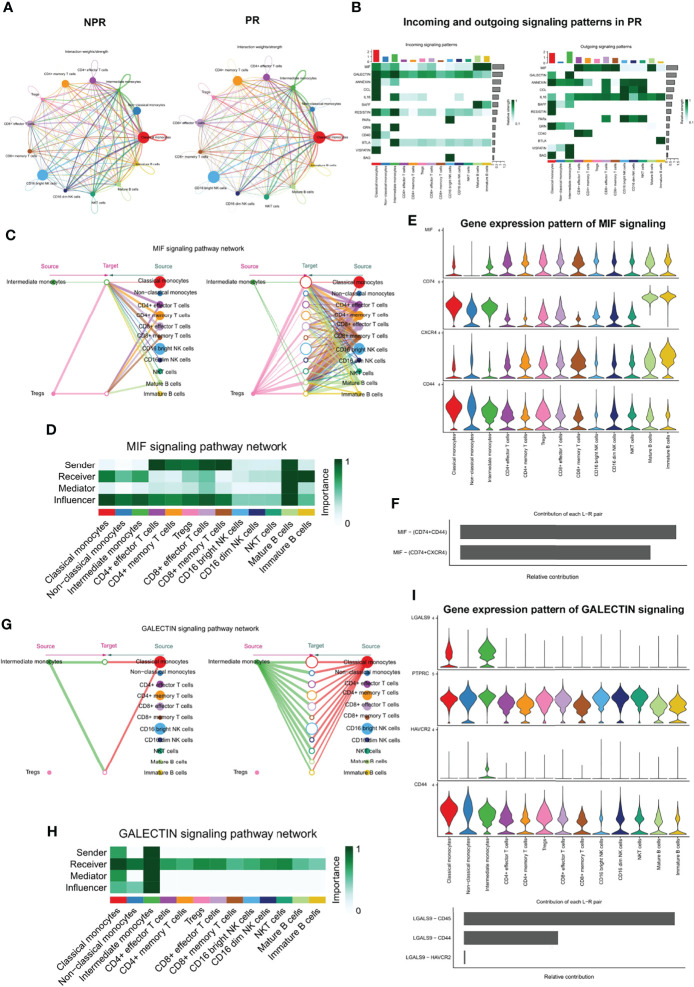
Complex intercellular communication network in the circulating immune cells in patients with AMI. **(A)** Network visualizing potential specific interactions in patients with PR and NPR, in which nodes are clusters and edges represent the number of significant ligand–receptor pairs. **(B)** Incoming and outcoming signaling pattern in patients with PR. **(C)** Hierarchical plot showing the inferred intercellular communication network for MIF signaling pathway. Left and right portions highlight the autocrine and paracrine signaling to intermediate monocytes, Tregs, and to other monocytes, T cell types, NK, and B cell types, respectively. **(D)** Heatmap showing the relative importance of each cell group for the four network centrality measures-based MIF signaling network. **(E)** Violin plot showing the expression distribution of signaling genes involved in the inferred MIF signaling network. **(F)** Relative contribution of each ligand–receptor pair to the overall communication network of MIF signaling pathway. **(G)** The inferred GALECTIN signaling pathway network. **(H)** The computed network centrality measures of GALECTIN signaling. **(I)** The expression distribution of signaling genes involved in the inferred GALECTIN signaling network. **(J)** Relative contribution of each GALECTIN ligand–receptor pair.

To systematically investigate interactions in circulating immune cells, we constructed a cell–cell communication network based on CellChat (20). The overall analysis of cell–cell communications between patients with PR and NPR was similar, so we focused on the intercellular communications in PR (**Figure 8A**). The top signaling pathways detected by CellChat included the MIF, GALECTIN, ANNEXIN, CCL, IL16, BAFF, RESISTIN, PARs, GRN, CD40, BTLA, VISFATIN, and BAG pathways (**Figure 8B**).

Macrophage migration inhibitory factor (MIF) is an important pro-inflammatory factor in atherosclerosis. It has been reported that MIF promotes atherogenic leukocyte recruitment and pathological inflammation by interacting with the chemokine receptors CXCR2 and CXCR4 (30, 31). Analysis of the network centrality of the deductive MIF signaling pathway showed that T-cell subtypes are the main sources of MIF ligands acting on monocytes and B cells (**Figures 8C, D**). Notably, mature B cells play an important role as the primary mediator of cell–cell communication. This suggests that the MIF signaling pathway in plaque rupture is complex and highly redundant, with a large proportion of monocytes targeted by multiple ligands. CellChat indicates that most MIF signaling interactions among immune cells are paracrine, with only mature B cells demonstrating significant autocrine signaling. Notably, two MIF ligand–receptor pairs, namely, CD74-CD44 and CD74-CXCR4, were the primary contributors to the MIF signaling pathway (**Figures 8E, F**), which played an important role in promoting atherosclerotic inflammation (30).

In contrast with MIF, CellChat analysis of the inferred GALECTIN signaling pathway revealed its very distinct, non-redundant structure with only one ligand (LGALS9) and classical and intermediate monocytes driving monocytes-to-monocytes and monocytes-to-other immune cells (**Figure 8G**). Network centrality analysis confirmed that monocyte populations are prominent influencers controlling communication (**Figure 8H**). Classical and intermediate monocytes were the primary GALECTIN signaling sources in both the autocrine and paracrine pathways, with LGALS9-CD45, LGALS9-CD44, and LGALS9-HAVCR2 ligand–receptor pairs driving the signaling (**Figures 8I, J**). These results reveal entirely different roles for MIF and GALECTIN pathways in plaque rupture.

## Discussion

Here we present a comprehensive single-cell resolution landscape of circulating immune cells in patients with PR and NPR. This large-scale project will advance the precise treatment of AMI, as it is difficult to distinguish between PR and NPR in clinical practice. The peripheral circulating immune environment of patients with AMI is more complicated and heterogeneous than previously recognized. Previous studies on peripheral circulating immune cells in patients with AMI were mainly based on flow cytometry to aggregate immune cells into major cell types (26, 32). Here, we analyzed 82,550 immune cells by single-cell RNA sequencing and identified 27 cell clusters and 6 immune cell subtypes, including monocytes, T cells, NK cells, B cells, megakaryocytes, and CD34^+^ cells. Some clusters (T cells and monocytes) are normally present at low levels under normal conditions but proliferate during the acute phase of AMI. We believe that these cell clusters are activated during the inflammatory process and contribute to the inflammatory severity of AMI.

AMI is reportedly a monocyte and T cells mediate inflammation (4, 33). Patients with PR exhibit a higher expression of pro-inflammatory genes in monocytes and demonstrate signs of T-cell activation (34). Previous studies have shown that monocytes are actively collected from the circulation into developing atherosclerotic plaques and differentiate into macrophages, eventually forming foam cells (34, 35). While previous studies have reported increased expression of circulating cytokines (36, 37), this study provides more insight into inflammatory genetic markers in circulating monocyte populations in patients with PR. Chemokine receptor CCL5 and the inflammatory factors TLR7 and CX3CR1 play a significant role in recruiting circulating monocytes and are all significantly expressed in both classical and non-classical monocytes in patients with PR (38, 39). In addition to the observed increased expression of pro-inflammatory cytokines, we further determined that leukocyte-endothelial adhesion genes such as ICAM1, ITGAL, and RUNX3 were significantly expressed in patients with PR. These are involved in promoting monocyte-endothelial adhesion. Interestingly, non-classical monocytes of patients with PR expressed more inflammatory proteins, such as TLR4, TNF, and CX3CR1. These data suggest that patients with PR not only enhance inflammatory cytokine release but also monocyte recruitment and innate immune response.

Nevertheless, patients with NPR mainly had intermediate monocytes and presented with distinct characteristics. Autopsy studies have indicated that plaque erosion was most frequent in those with hypertriglyceridemia or diabetes, females, and the young (40, 41). Lesions in plaque erosion were rich in proteoglycans and glycosaminoglycans, with relatively few macrophages and T lymphocytes, while PR contained numerous necrotic, lipid-rich cores (42). Compared with PR, individuals with AMI and NPR defined by OCT had higher levels of systemic myeloperoxidase (MPO), similar to coronary thrombus (43).

In our study, we found that intermediate monocytes were predominantly in patients with NPR and expressed more neutrophil activation genes, such as FPR2, MMP9, and CLEC4D, which may contribute to neutrophil activation and neutrophil degranulation, ultimately leading to plaque erosion. In this study, T cells were divided into five clusters, namely, CD4^+^ effector T cells, CD4^+^ memory T cells, Tregs, CD8^+^ memory T cells, and CD8^+^ effector T cells. Although T cells were heterogeneous, their cell percentage did not differ significantly between the two groups. Previous studies have shown that CD8^+^ effector T cells secrete various effector molecules such as GZMB, GNLY, and PRF1 (26), leading to plaque erosion through endothelial cell apoptosis and desquamation, which is consistent with our study. We also found that CD4^+^ effector T cells may contribute to PR by producing a range of cytokines and inflammation-related genes. Another novel finding in this study is that CD8^+^ effector T cells may promote angiogenesis and wound healing in NPR patients by the expression of CXCR4, B4GALT1, and TNFAIP3, providing an explanation for faster healing in patients with plaque erosion (27).

NK and B cells also showed distinct characteristics between patients with PR and NPR. NK cells are characterized by increased cytokine production and promote the migration and activation of inflammatory cells in PR patients. B cells exhibit similar characteristics to NK cells. Mature B cells primarily contribute to the migration of inflammatory cells and cytokine production in PR patients. In this study, our data revealed the significant role of metabolism-related signaling pathways in patients with NPR, while PR patients showed significantly enriched inflammatory cell recruitment and immune response activation.

Our pseudo-time analysis clearly describes the development of different cell types, indicating that cells are mainly associated with metabolism in the naive stage and the activation of inflammatory cells and cytokine production in the late stage. As expected, in the initial stage, classical and intermediate monocytes presented as translational initiation and neutrophil activation involved in the immune response. Then, these cells gradually transformed into non-classical monocytes with a toll-like receptor signaling pathway and activation of the innate immune response, which presented as a highly pro-inflammatory state. Pseudo-time analyses of other cell types showed similar characteristics.

Similarly, SCENIC analysis of CD8^+^ effector T cells and CD16^bright^ NK cells identified TFs that can enhance their cytotoxicity in the microenvironment of AMI. This finding lays the foundation for further exploration of the role of TFs in immune cell pathology. Cell–cell interactions suggest that immune cells communicate extensively with each other. We found that intercellular communication was altered in patients with PR. The significant signaling pathways detected by CellChat included the MIF, GALECTIN, ANNEXIN, CCL, IL16, BAFF, RESISTIN, PAR, GRN, CD40, BTLA, VISFATIN, and BAG pathways. Analysis of the network centrality of the inferred MIF signaling pathway showed that several T cell populations were the main sources of MIF ligand action on monocytes and B cells. We also analyzed the GALECTIN signaling network, with only one ligand (LGALS9) and classical and intermediate monocytes driving monocytes-to-monocytes and monocytes-to-other immune cells. These results are consistent with the known remarkable role of monocytes in initiating inflammation during plaque rupture and driving the activation of resident macrophages through MIF and GALECTIN signaling (44–47).

In conclusion, classical and non-classical monocytes constitute the cornerstone of the inflammatory state noted in patients with AMI. Intermediate monocytes and CD8^+^ effector T cells may mainly by activating neutrophils, promoting degranulation of neutrophils and endothelial death then leading to plaque erosion, while CD4^+^ effector T cells and non-classical monocytes may accelerate the progression of plaque rupture through proinflammatory activation. This study demonstrates that the circulating immune cells of patients with plaque rupture exhibit highly pro-inflammatory characteristics. These data provide a detailed landscape of the immunological network of circulating cells in patients with AMI. These findings elucidate the pathological process of inflammatory progression in patients with AMI and identify potential therapeutic targets. Although this is a large patient-specific single-cell RNA sequencing study, the enrolled sample is relatively small because of the high cost of the new technologies involved. Because of the small sample size and lack of long-term follow-up data, it is impossible to identify the effect of the newly defined immune cell markers on prognosis. Additionally, re-clustering of cell types may exaggerate statistical differences between the cell clusters. Further validation techniques, such as flow cytometry, are necessary to validate the observed markers of immune disorders and to clarify possible pathogenesis roles in a larger population of patients with AMI.

## Data Availability Statement

The name of the repository and accession number can be found below: Gene Expression Omnibus, GSE269269.

## Ethics Statement

The ethics committee of Shanghai Tongji Hospital reviewed and approved the study protocol. The research is in line with the Declaration of Helsinki. The patients/participants provided their written informed consent to participate in this study. Written informed consent was obtained from the individual(s) for the publication of any potentially identifiable images or data included in this article.

## Author Contributions

JQ and FC performed the data analysis. JQ, YL, ZY, KD, YiY, JT, YuY, JC, FC, and XL performed the intravascular imaging studies. JQ, YG, YL, ZY, FC, and XL maintain responsibility for clinical data. HL, GZ, YuY, HD, and DY performed the experiment. JQ, YG, FC, and XL designed the study. JQ wrote the manuscript with critical input of FC and XL. All authors listed have made a substantial, direct, and intellectual contribution to the work and approved it for publication.

## Funding

This study was supported by the National Natural Science Foundation of China (Grant Nos. 81670403 and 81370390), the Shanghai Science and Technology Committee (Nos. 18411950300, 19XD1403300, and 19411963200), and the Shanghai Municipal Health Commission (No. 2019LJ10).

## Conflict of Interest

The authors declare that the research was conducted in the absence of any commercial or financial relationships that could be construed as a potential conflict of interest.

## Publisher’s Note

All claims expressed in this article are solely those of the authors and do not necessarily represent those of their affiliated organizations, or those of the publisher, the editors and the reviewers. Any product that may be evaluated in this article, or claim that may be made by its manufacturer, is not guaranteed or endorsed by the publisher.
