# Corrigendum: Single-cell RNA sequencing of peripheral blood mononuclear cells from acute myocardial infarction

**DOI:** 10.3389/fimmu.2024.1462970

**Published:** 2024-08-07

**Authors:** Jun Qian, Yanhua Gao, Yan Lai, Zi Ye, Yian Yao, Keke Ding, Jing Tong, Hao Lin, Guoqi Zhu, Yunan Yu, Haoran Ding, Deqiang Yuan, Jiapeng Chu, Fei Chen, Xuebo Liu

**Affiliations:** Department of Cardiology, Tongji Hospital, Tongji University School of Medicine, Shanghai, China

**Keywords:** plaque rupture, plaque erosion, single-cell RNA sequencing, acute myocardial infarction, inflammatory

**Incorrect Data Availability Statement**

In the published article, there was an error in the Data Availability statement. The data uploaded from the original URL cannot be browsed, we have re-uploaded the data to the GEO database. The correct Data Availability statement appears below.

**Data Availability Statement**

The name of the repository and accession number can be found below: Gene Expression Omnibus, GSE269269.

The authors apologize for this error and state that this does not change the scientific conclusions of the article in any way. The original article has been updated.

