# Supplementary material for: Single-Cell RNA Sequencing of Peripheral Blood Mononuclear Cells From Acute Myocardial Infarction
Source: Front Immunol. 2022 Jun 29;13:908815. doi: 10.3389/fimmu.2022.908815 (PMC9278132; doi:10.3389/fimmu.2022.908815)
Supplement: Supplementary file 1 [file DataSheet_1.doc]

| **Supplement Table1. Baseline characteristics of patients with plaque rupture and without rupture** | | | |
| --- | --- | --- | --- |
| Variables | NPR(n=5) | PR(n=5) | p value |
| Age | 59.8±15.8 | 65.8±6.5 | 0.46 |
| Male | 5 | 4 | 0.29 |
| BMI, kg/m2 | 25.3±1.2 | 24.3±1.5 | 0.28 |
| ACS type |  |  | 0.11 |
| STEMI | 5 | 3 |  |
| NSTEMI | 0 | 2 |  |
| Hypertension | 2 | 2 | 0.99 |
| Diabetes | 0 | 1 | 0.29 |
| Smoke | 4 | 2 | 0.20 |
| cTnI, ng/ml | 15.9±34.7 | 9.4±13.9 | 0.71 |
| CK-MB, u/L | 66.6±128.9 | 110.9±96.7 | 0.56 |
| BUN, mmol/L | 5.4±1.0 | 5.3±1.0 | 0.90 |
| eGFR, ml/min/1.73m2 | 114.3±59.5 | 94.2±13.8 | 0.48 |
| D-Dimer, mg/L | 1.2±2.2 | 0.9±0.6 | 0.74 |
| Glucose, mmol/L | 6.4±0.7 | 5.7±0.7 | 0.12 |
| GHb | 6.1±0.5 | 7.2±3.0 | 0.42 |
| Tc, mmol/L | 4.9±1.1 | 4.3±1.7 | 0.56 |
| LDL-C, mmol/L | 3.4±1.1 | 3.0±1.3 | 0.64 |
| Uric acid, mmol/L | 378.2±54.5 | 338.6±68.6 | 0.34 |
| Hs-CRP, mg/L | 7.9±3.8 | 16.6±18.2 | 0.33 |
| NT-Pro-BNP, ng/L | 935.3±265.0 | 764.1±748.8 | 0.64 |
| Abbreviations：NPR, non-plaque rupture; PR, plaque rupture; BMI, body mass index; ACS, acute coronary syndrome; STEMI, ST-segment elevation myocardial infarction; NSTEMI, non-ST-segment elevation myocardial infarction; cTnI, cardiac troponin I; CK-MB, Creatine Kinase Isoenzyme-MB; BUN, blood urea nitrogen; eGFR, estimated glomerular filtration rate; GHb, glycosylated hemoglobin; Tc, total cholesterol; LDL-C, low density lipoprotein cholesterol; Hs-CRP, high-sensitivity C-reactive protein; NT-Pro-BNP, N-terminal pro-brain natriuretic peptide.  **Supplement Table 2. Cell numbers and sequencing information of each patient.**  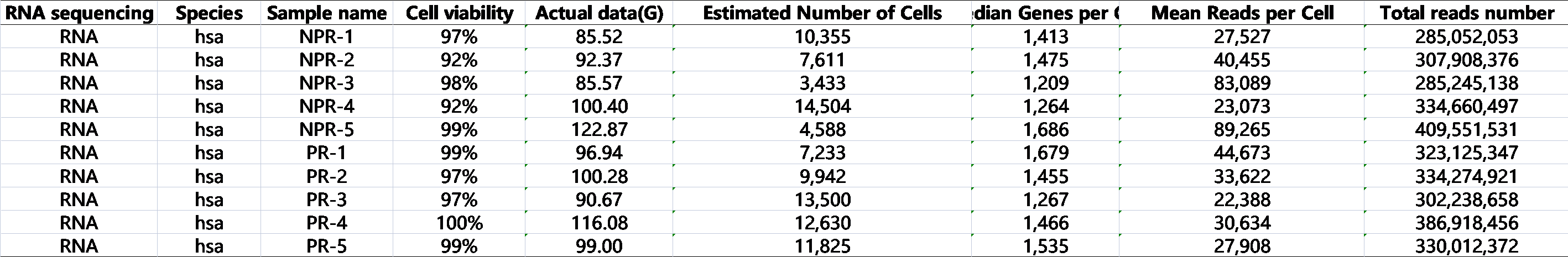 | | | |

**Supplement Figure Legend**

**Supplement Figure 1.**


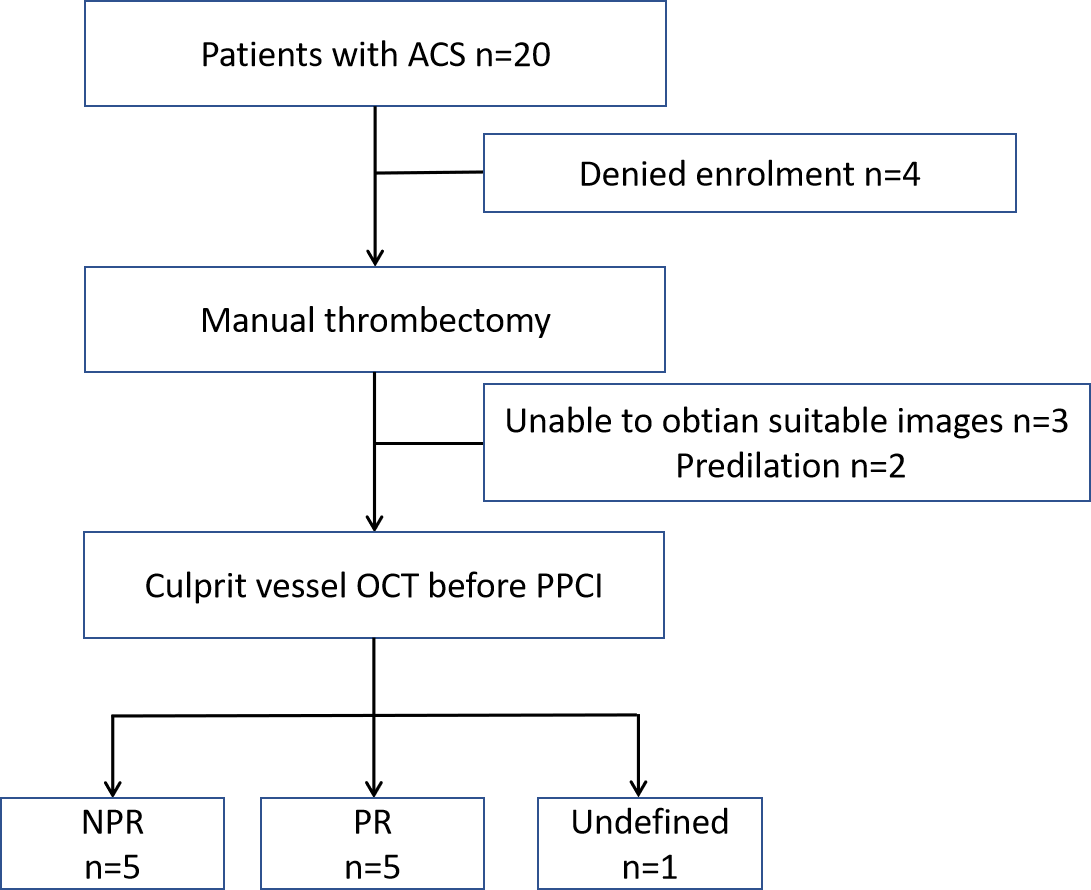


Supplement Figure 1. Study chart flow. Abbreviations: ACS, acute coronary syndrome. OCT, optical coherence tomography. PPCI, primary percutaneous coronary intervention. NPR, non-plaque rupture. PR, plaque rupture.

**Supplement Figure 2.**


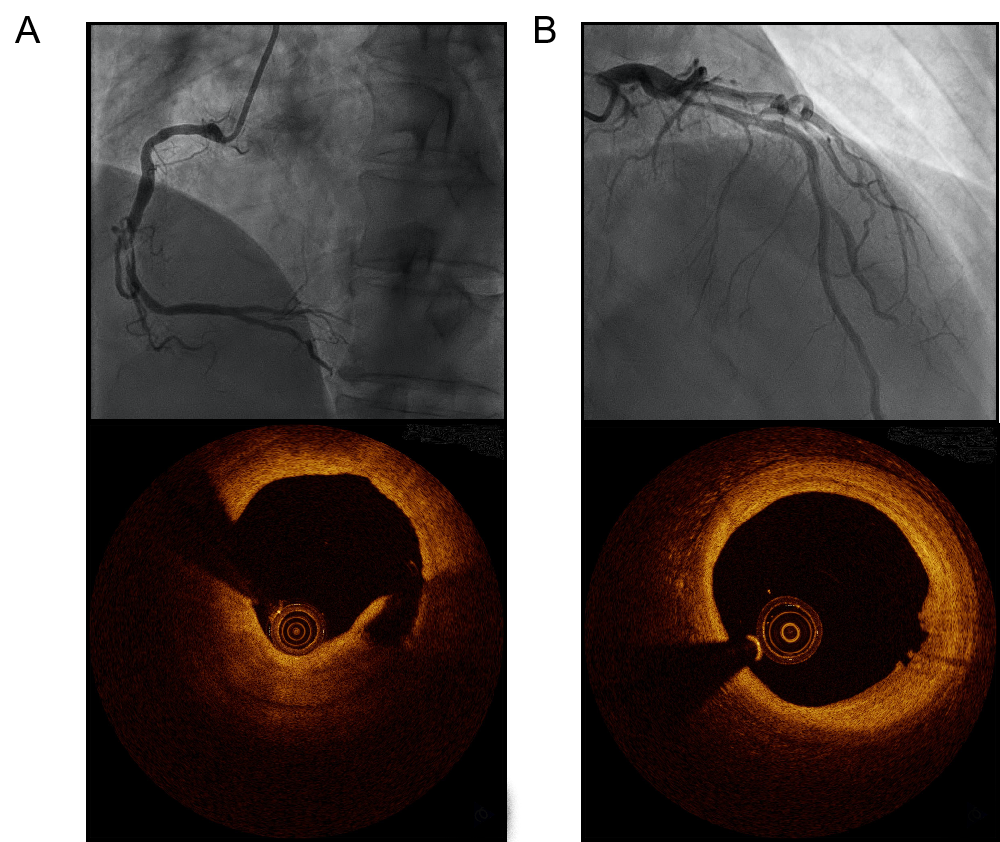


Supplement Figure 2. OCT images of typical plaque rupture and without plaque rupture. (A) Representative OCT images of plaque rupture. (B) Representative OCT images of non-plaque rupture.

**Supplement Figure 3.**


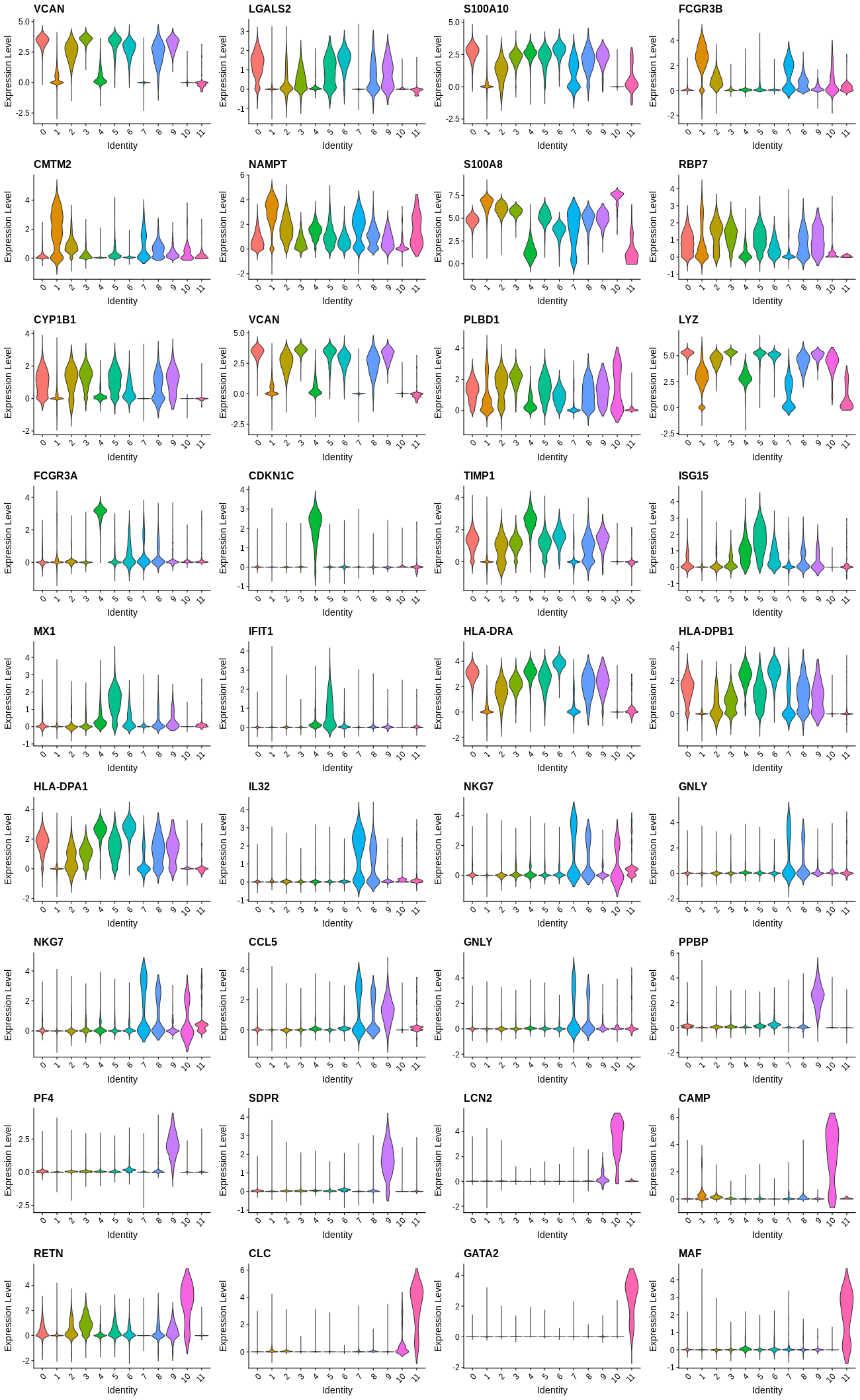


Supplement Figure 3. Violin plot of marker gene expression of each cluster in monocytes.

**Supplement Figure 4.**


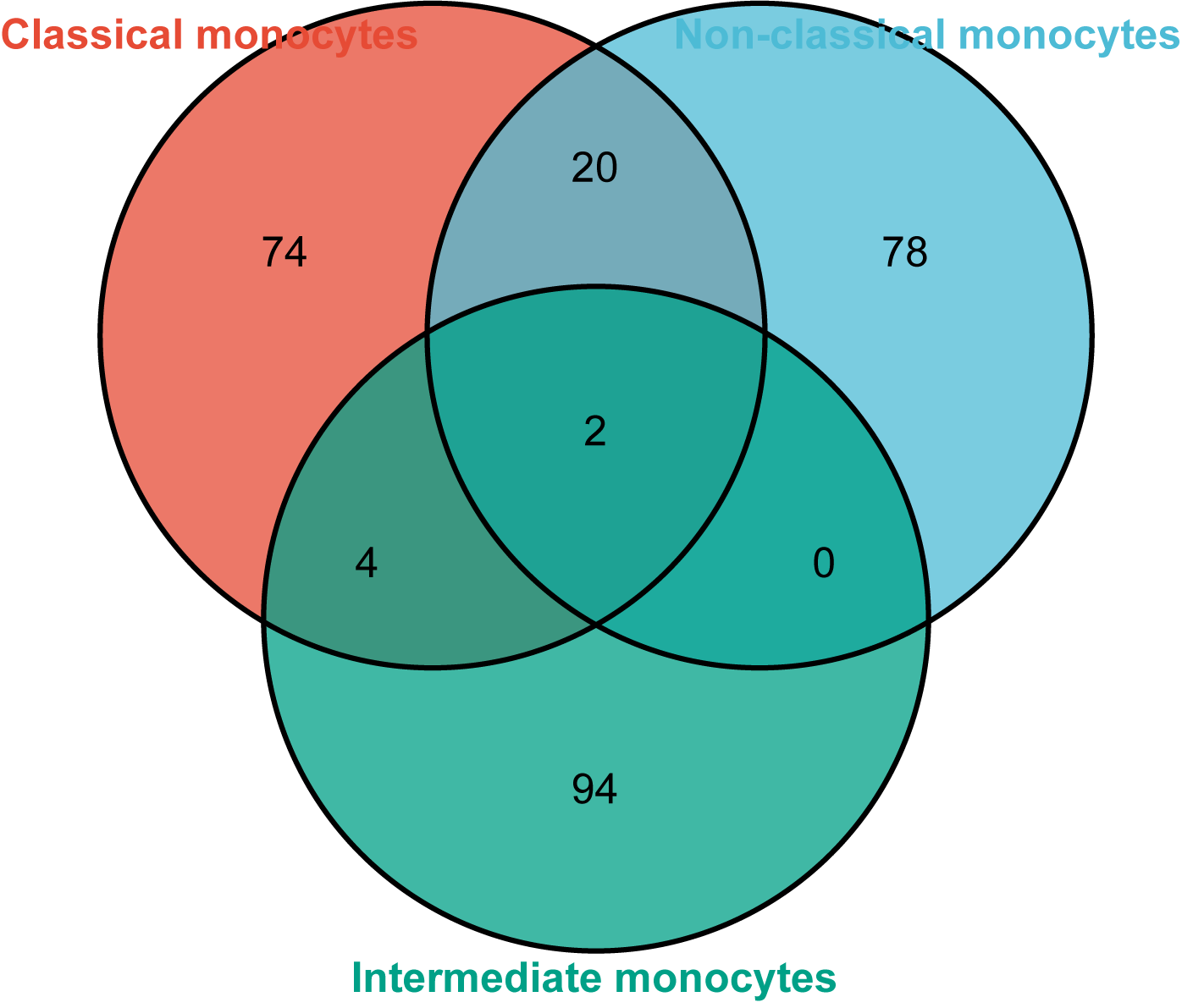

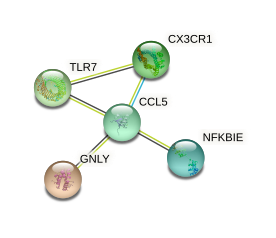


A B

Supplement Figure 4. A. Venn diagrams show the number of common genes of three monocyte types. B. Up-regulated hub gene in both classical and non-classical monocytes.

**Supplement Figure 5.**


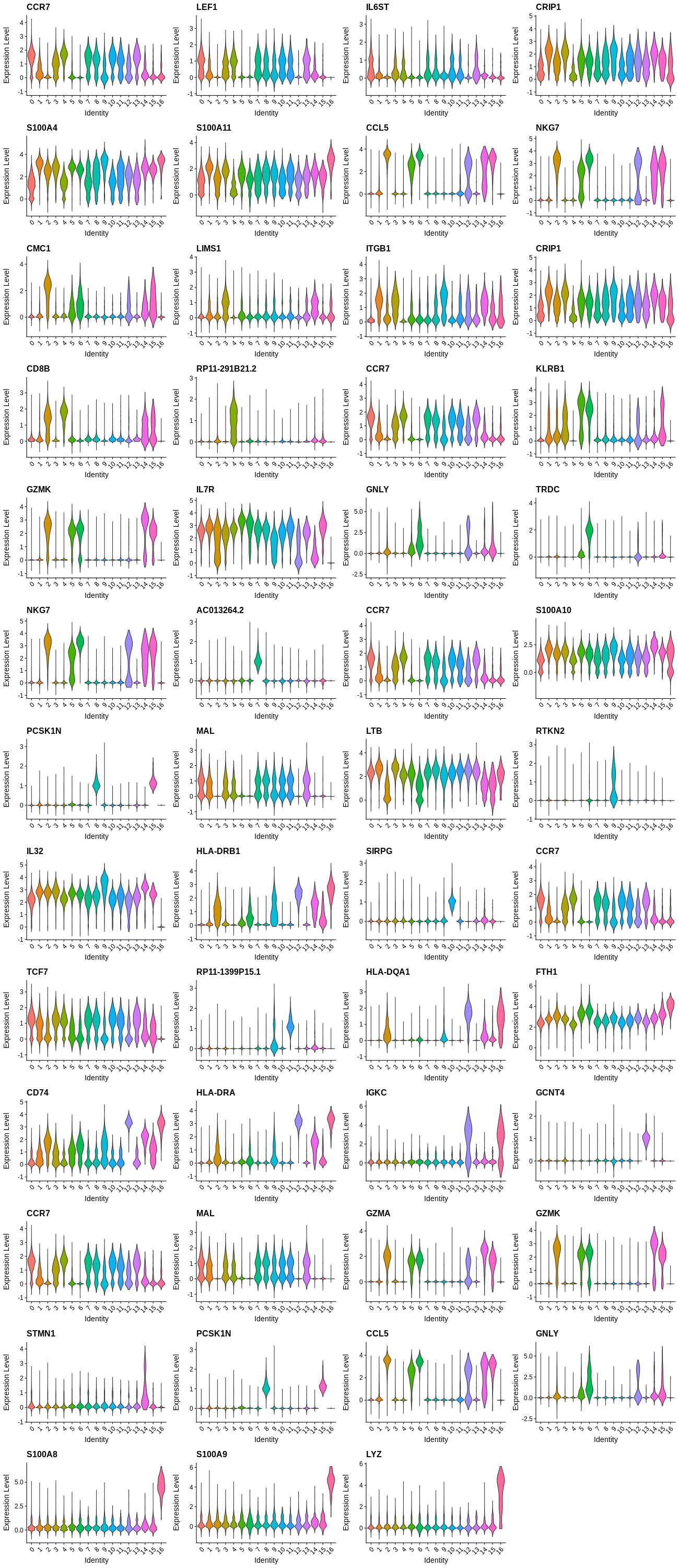


Supplement Figure 5. Violin plot of marker gene expression of each cluster in T cells clusters.

**Supplement Figure 6.**


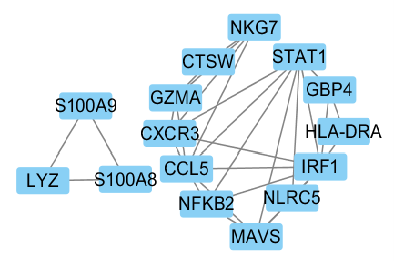

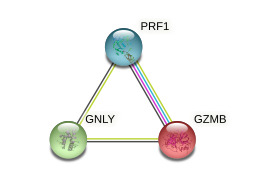


A B

Supplement Figure 6. A. Up-regulated DEGs in patients with PR derived from CD4^+^ effector T cells. B. Up-regulated DEGs in patients with NPR from CD8^+^ effector T cells.

**Supplement Figure 7.**


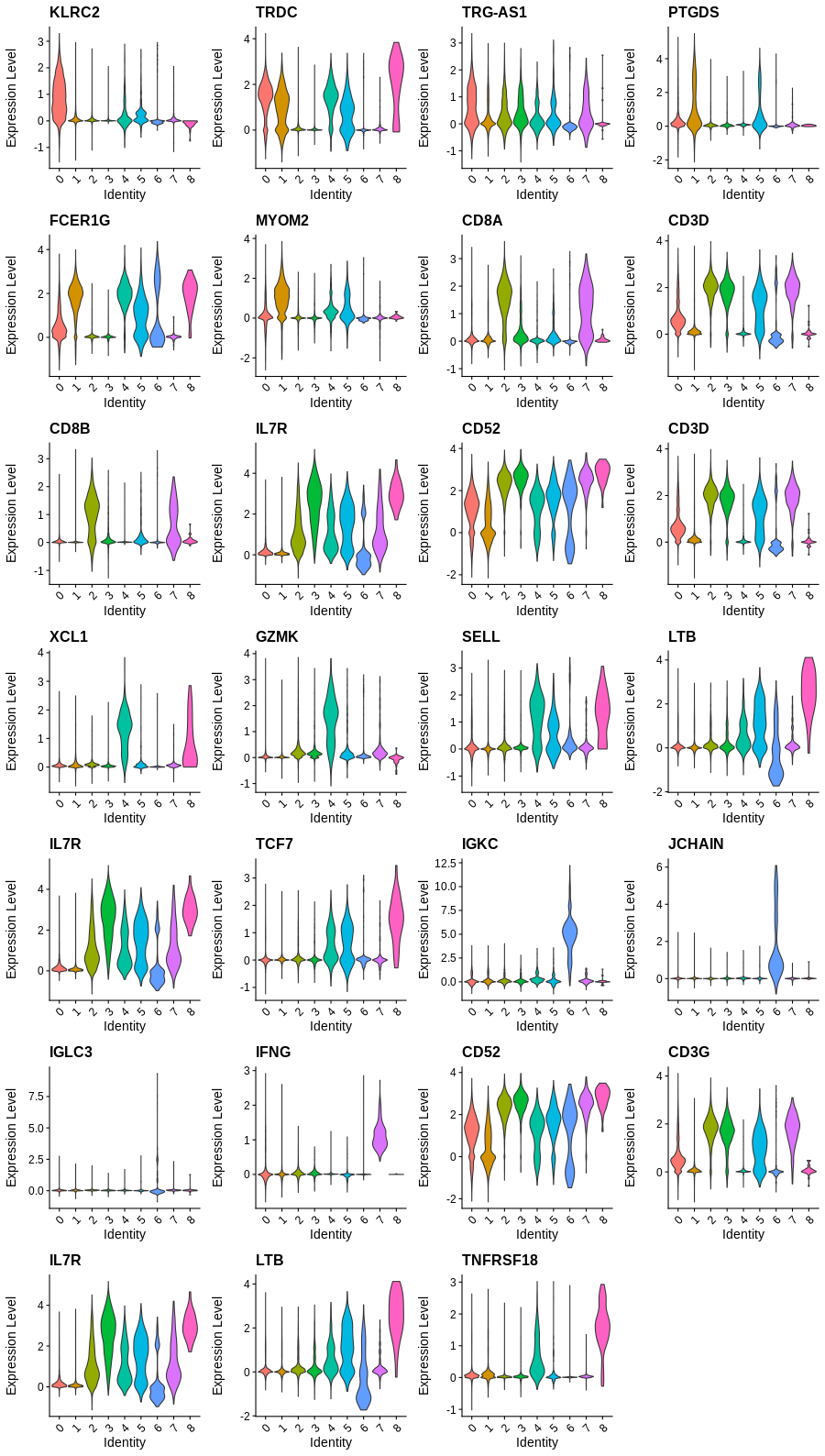


Supplement Figure 7. Violin plot of marker gene expression of each cluster in NK cells clusters.

**Supplement Figure 8.**

**All NK cells GO in NPR**


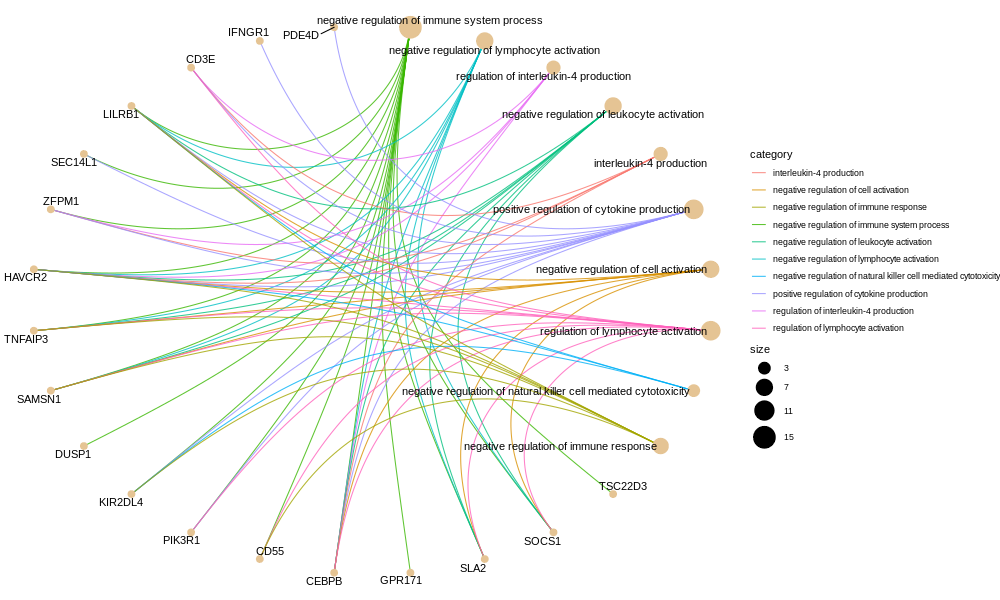


A

**CD16dim NK cells GO in NPR**


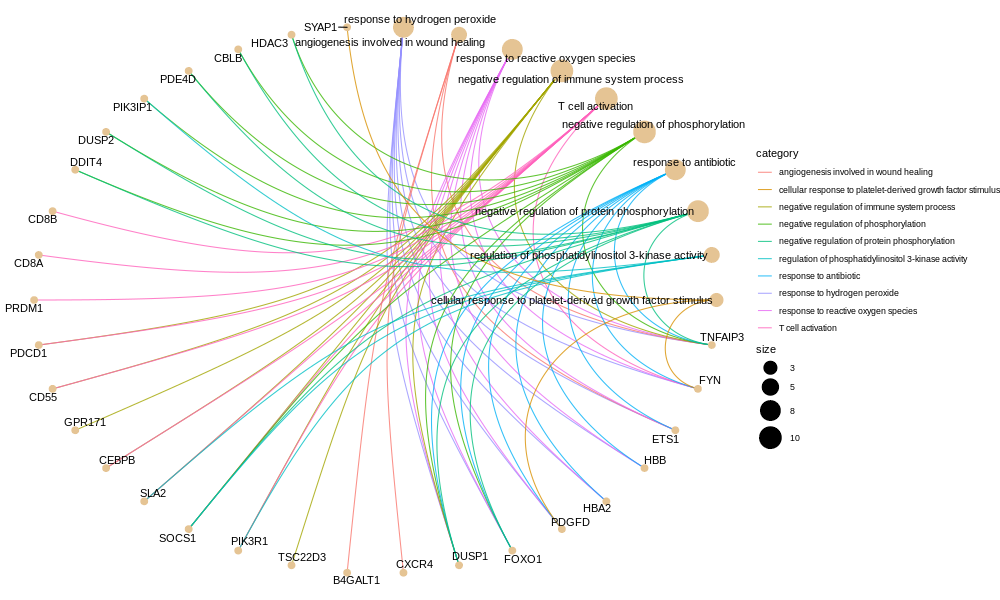


B

**NKT cells GO in NPR**


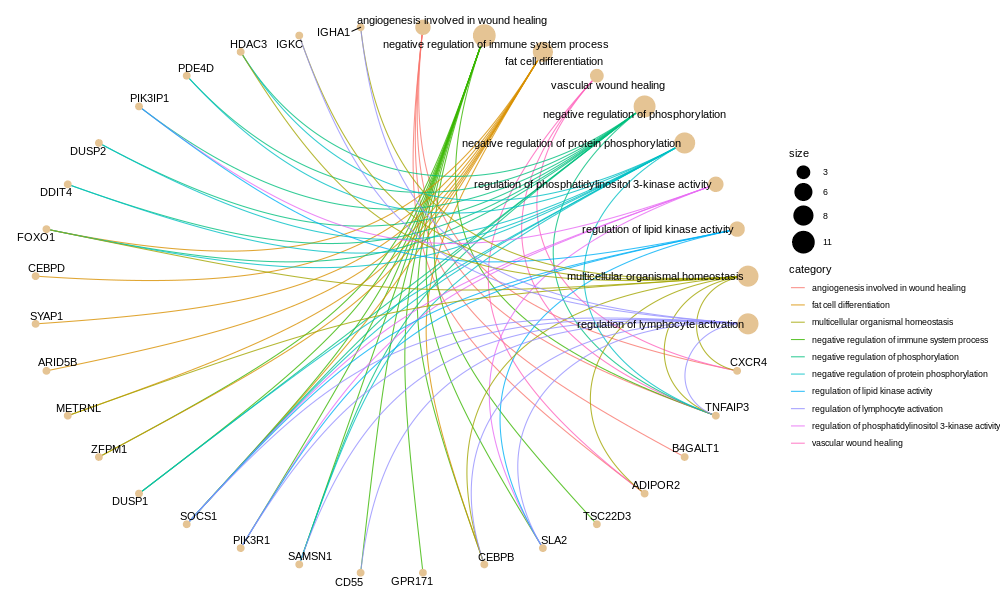


C

Supplement Figure 8. A. GO analysis of all NK cells in patients with NPR. B. GO analysis of CD16^dim^ NK cells in patients with NPR. C. GO analysis of NKT cells in patients with NPR.

**Supplement Figure 9.**


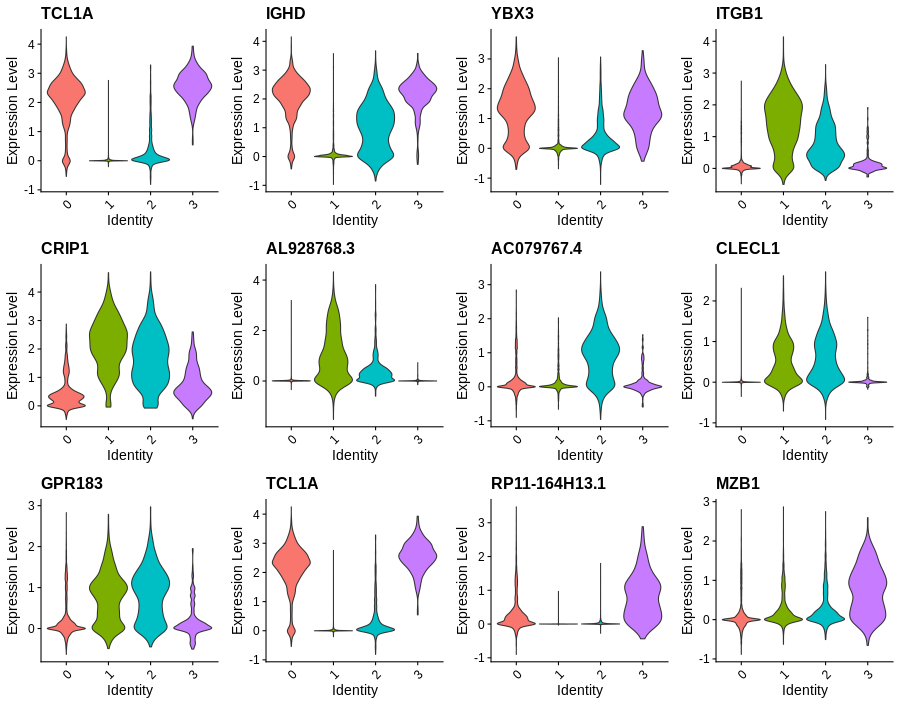


Supplement Figure 9. Violin plot of marker gene expression of each cluster in B cells clusters.

**Supplement Figure 10.**

**Mature B cells GO in NPR**


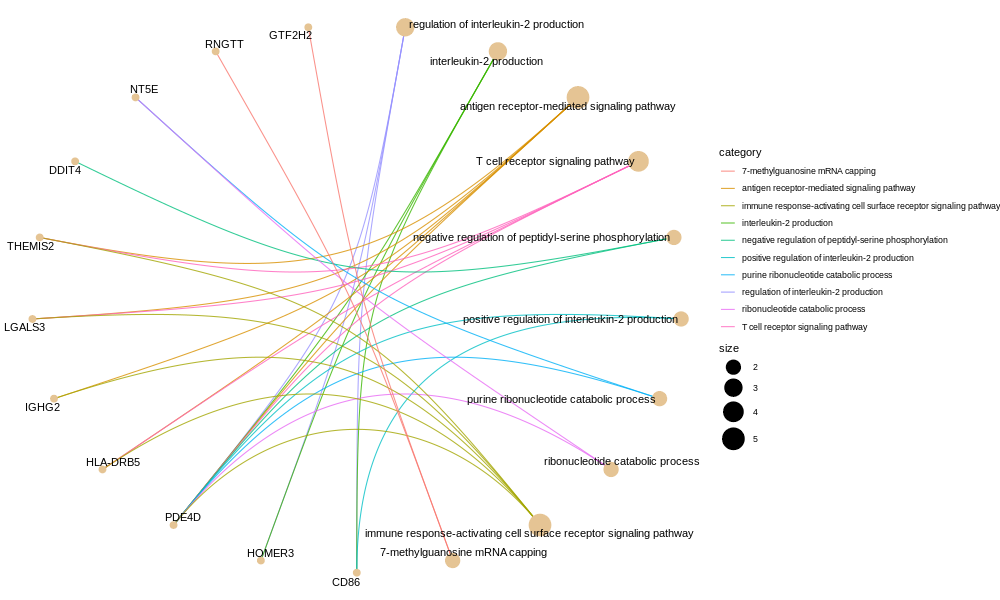


Supplement Figure 10. GO analysis of mature B cells in patients with NPR.
